# Suppression of *Streptococcus mutans* Biofilm Formation and Gene Expression by PRG Barrier Coat: A Molecular and Microscopic Study for Preventing Dental Caries

**DOI:** 10.3290/j.ohpd.b4928623

**Published:** 2024-02-02

**Authors:** Haruka Nishimata, Yoko Kamasaki, Kyoko Satoh, Risako Kinoshita, Keisuke Omori, Tomonori Hoshino

**Affiliations:** a Assistant Professor, Department of Paediatric Dentistry, Nagasaki University Graduate School of Biomedical Sciences, Nagasaki, Japan. Conceived the idea and experimental design, collected the data, wrote the manuscript, analysed the data, performed statistical evaluation.; b Director, Nagasaki Dental Health Center, Nagasaki, Japan. Conceived the idea and experimental design, collected the data, analysed the data, performed statistical evaluation.; c Visiting Researcher, Department of Pediatric Dentistry, Nagasaki University Graduate School of Biomedical Sciences, Nagasaki, Japan. Conceived the idea and experimental design, collected the data.; d Dentist and PhD Student, Department of Pediatric Dentistry, Nagasaki University Graduate School of Biomedical Sciences, Nagasaki, Japan. Proofread the manuscript.; e Medical Staff, Department of Clinical Oral Oncology, Nagasaki University Graduate School of Biomedical Sciences, Nagasaki, Japan. wrote the manuscript, analysed the data, performed statistical evaluation, proofread the manuscript.; f Professor, Division of Paediatric Dentistry, Department of Human Development and Fostering, Meikai University School of Dentistry, Sakado, Japan. Conceived the idea and experimental design, collected the data, wrote and proofread the manuscript.

**Keywords:** biofilm, PRG Barrier Coat, reverse-transcription polymerase chain reaction (RT-PCR), scanning electron microscopy (SEM), *Streptococcus mutans*

## Abstract

**Purpose:**

This study aimed to investigate the inhibitory effect of a PRG Barrier Coat on biofilm formation and structure by *Streptococcus mutans* and propose an effective method for preventing dental caries.

**Materials and Methods:**

*Streptococcus mutans* MT8148 biofilms were obtained from hydroxyapatite disks with and without a PRG Barrier Coat. Scanning electron microscopy (SEM) was used to observe the 12- and 24-h-cultured biofilms, while reverse-transcription polymerase chain reaction (qRT-PCR) was used to quantify caries-related genes. Biofilm adhesion assessments were performed on glass. Statistical analysis was performed using a two-sample t-test.

**Results:**

A statistically significant difference in *Streptococcus mutans* biofilm adhesion rate was observed between the control and PRG Barrier Coat-coated samples (p < 0.01). However, there was no statistically significant difference in total bacterial count or biofilm volume (p > 0.05). SEM revealed that the PRG Barrier Coat inhibited biofilm formation by *Streptococcus mutans*. Real-time RT-PCR revealed that the material restricted the expression of genes associated with caries-related biofilm formation. However, the suppression of *gtf*D and *dex*B differed from that of other genes.

**Conclusion:**

PRG Barrier Coat suppressed biofilm formation by *Streptococcus mutans* by inhibiting the expression of insoluble glucan synthase, which is associated with primary biofilm formation. The material also affected gene expression and altered the biofilm structure. Tooth surface-coating materials, such as PRG Barrier Coat, may improve caries prevention in dental practice.

Preventing dental caries is a critical aspect of dental care. Effectively reducing or eliminating caries risk factors, including a cariogenic diet, bacterial presence, host factors, and prolonged exposure to dietary sugars in the oral environment, is crucial for successful caries prevention. Although dentists, especially paediatric dentists, endeavor to discourage unhealthy habits, such as frequent consumption of sugary snacks between meals, in children at high risk for caries, these interventions frequently prove ineffective.

The glucan produced by *Streptococcus mutans* forms a biofilm that causes caries. *S. mutans* produces three types of glucosyltransferases, GTF-B, C, and D, which are important for cellular adhesion.^1,6,13^ Additionally, sugar-metabolising enzymes such as dextranase and galactosidase are related to acid production, which demineralises the enamel. Therefore, the inhibition of biofilm formation by *S. mutans* is effective in preventing dental caries.

In previous studies, the antimicrobial activity of dental materials has been evaluated for the purpose of caries prevention.^7,11,16^ However, no material that can reliably prevent caries has been established. Surface pre-reacted glass-ionomer (S-PRG) filler is an active ingredient that can release and recharge fluoride ions. Additionally, the S-PRG filler can release five other active ions, strontium, silicate, sodium, borate, and aluminum. It also has a modulating effect on acidic conditions, which helps maintain a close-to-neutral pH when it comes into contact with water or acidic solutions.^4,9,17^ PRG Barrier Coat (Shofu; Kyoto, Japan) is a fluoride-releasing coating that suppresses dentin hypersensitivity. Sai et al^11^ reported that PRG Barrier Coat protects roots from demineralisation in vitro, based on micro-CT scanning and scanning electron microscopy (SEM) observations. Örtengren et al^14^ reported that PRG Barrier Coat significantly reduced plaque accumulation in healthy adults over a 60-day period, but did not affect pH stabilisation or bacterial composition.

However, no detailed in-vitro studies have examined whether PRG Barrier Coat suppresses biofilm formation by the caries-related enzymes of *S. mutans*. The purpose of this study was to determine the inhibitory effect of PRG Barrier Coat on caries-related enzymes, as well as the biofilm formation and structure of *S. mutans*, using molecular biological techniques and SEM.

## Materials and Methods

### Bacterial Strain and Culture Condition

This study utilised *S. mutans* MT8148 isolated from the oral cavities of Japanese children. The bacteria were cultured in Brain Heart Infusion (BHI) broth (Difco Laboratories; Detroit, MI, USA) under anaerobic conditions for 16 h at 37°C.^2^

### Investigation of the Effect of Suppressing Adhesion to a Smooth Surface by PRG Barrier Coat

Precultures of *S. mutans* were inoculated in a BHI medium containing 1% sucrose and cultured in a PRG Barrier Coat-treated glass tube tilted at a 30-degree angle for 12 h at 37°C. The adherence rate was calculated by measuring the amount of biofilm that adhered to the tube wall. The effect of PRG Barrier Coat on suppressing adhesion to the smooth surface and biofilm production was investigated based on turbidity measured using Genesys 10vis (Thermo Fisher Scientific; Waltham, MA, USA) and the adhesion rate. The total bacterial count was calculated as the sum of the OD_600_ values of the adherent and non-adherent bacteria. The adherence rate was determined by calculating the percentage: (OD_600_ value of adherent bacteria / OD_600_ value of total bacterial count). The means and standard deviations of three experiments were used as the analysis results.

### Biofilm Formation on the Hydroxyapatite Disk

Hydroxyapatite (HA) disks (Cellyard Pellet, Pentax; Tokyo, Japan) were sterilised and prepared with/without a coating of PRG Barrier Coat on one side. The samples without PRG Barrier Coat served as the control, and the samples with PRG Barrier Coat applied comprised the coated samples. These disks were placed at the bottom of a 6-well plate, filled with a culture medium of BHI containing 1% sucrose, and maintained for 12 and 24 h at 37°C. Then the biofilm on the surface of the disks was analysed.

### SEM Observation of In-Vitro Biofilms

HA disks were collected and subjected to fixation, dehydration, immersion in hexamethyldisilazane, drying, and evaporation by gold-sputter coating before being mounted on specimen tables.^20^ The method proposed by Weber et al^21^ was partially modified, and 60 mM HEPES buffer was used for the rinse. The surface properties and cross-sections of each sample were observed using a Hitachi S-3500 SEM (Hitachi; Tokyo, Japan).

### Real-time Reverse-Transcription Polymerase Chain Reaction (Real-Time RT-PCR)

Biofilms on the HA disks were collected for total RNA extraction after washing with PBS (pH 7.4) to remove the deposited *S. mutans* cells. RNA was extracted from the biofilm pellets using Cury’s method^3^ with some modifications. Briefly, the collected biofilm pellets were transferred to 15-ml Falcon tubes and centrifuged at 4°C, 10,000 × g, for 5 min. The supernatant was discarded, and the cells were washed with 5 ml PBS and centrifuged as described above. The recovered pellet was suspended in 100 μl of 1 mg/ml lysozyme solution and frozen at -80°C for 5 min. After thawing, 5 μl of 10 mg/ml Proteinase K and 5 μl of 100 U/L mutanolysine were added and let react at 50°C for 1 h. Total RNA was extracted from the reaction solution and purified using NucleoSpin RNA (Takara; Shiga, Japan).

In the first step in the two-step RT-PCR method, cDNA was synthesised from 20-µl samples of purified RNA using the cDNA Reverse Transcription Kit and RNA to cDNA EcoDryTM PreMix (Takara Bio; Shiga, Japan), which contained random hexamers and reverse transcriptase. RT was performed according to the manufacturer’s protocol for RNA-to-cDNA EcoDryTM PreMix.

The resulting cDNA was used as the template for PCR. Primers for *gtf* qRT-PCR were designed based on the paper by Fujiwara et al.^5^ Primers to amplify *gap*C, *dex*, and *lac*G were designed by extracting these gene sequences from the genome information of *S. mutans* UA159 and using ABI Prism Primer Express TM Version 2.0 (Thermo Fisher Scientific) (Table 1). This step was performed using the SYBR Green PCR Master Mix. PCR amplification and detection were performed according to the protocol provided along with the Applied Biosystems 7500 Real-Time PCR System.

**Table 1:** PCR primers used to amplify the 16S rRNA, *gap*C, *gtf*B, *gtf*C, *gtf*D, *dex*A, *dex*B, and *lac*G gene sequences

Target gene	Primer name	Primer sequence	Annealing temp. (ºC)	Expected size (bp)
16S rRNA	SMrrn-F	5′-CTCAGGCGCAAAAAGATGG-3′	60	81
	SMrrn-R	5′-ATTTCCCTGCAATTTCAAGACC-3′		
*gap*C	SMgapdh-F	5′-AGCTGAACGTGATCCAGAACAG-3′	60	78
	SMgapdh-R	5′-AAAGAAGCCAGTTGCTTCAAGAA-3′		
*gtf*B	B442-F	5′-AGCAATGCAGCCAATCTACAAAT-3′	60	98
	B537-R	5′-ACGAACTTTGCCGTTATTGTCA-3′		
*gtf*C	C236-F	5′-CTCAACCAACCGCCACTGTT-3′	60	90
	C326-R	5′-GGTTTAACGTCAAAATTAGCTGTATTAGC-3′		
*gtf*D	D434-F	5′-CACAGGCAAAAGCTGAATTAACA-3′	60	83
	D514-R	5′-GAATGGCCGCTAAGTCAACAG-3′		
*dex*A	dexA-F	5′-CTGACAACTGCGGCCATTG-3′	60	81
	dexA-R	5′-ACCACCCCCATCATTAGGATT-3′		
*dex*B	dexB-F	5′-CACGTGAGCATCCAGACAGTTC-3′	60	81
	dexB-R	5′-CACCGAAAATAGATTCCAAATCATT-3′		
*lac*G	lacG-F	5′-TCCAATCCCACCACAACATGA-3′	60	102
	lacG-R	5′-TTGGCAGAGCATGAACCACAC-3′		

PCR: polymerase chain reaction.

The expression of *gtf*, *dex*, and *lac*G was determined using real-time RT-PCR based on the comparative Ct method. The activity of *S. mutans* cells in the glucan biofilm and the effect of the PRG Barrier Coat on suppressing gene expression were evaluated by comparing the expression of biofilm-related genes and 16S rRNA between the control and coated groups when the internal standard was *gap*C.

### Statistical Analysis

Each experiment was performed in triplicate. The distribution of measurements obtained in the experiments examining the effect of PRG barrier coatings on the inhibition of biofilm deposition on smooth surfaces was tested using the Shapiro-Wilk test. As the data were normally distributed, they were analysed using a parametric two-sample t-test. Statistical analyses were performed using R Commander (version 4.2.2; R Core Team; Vienna, Austria) and IBM SPSS software v 24.0 (IBM; Armonk, NY, USA), and a two-tailed p-value < 0.05 was considered a statistically significant difference.

## Results

### Inhibitory Effects of PRG Barrier Coat on Adhesion to a Smooth Surface

**Fig 1:**
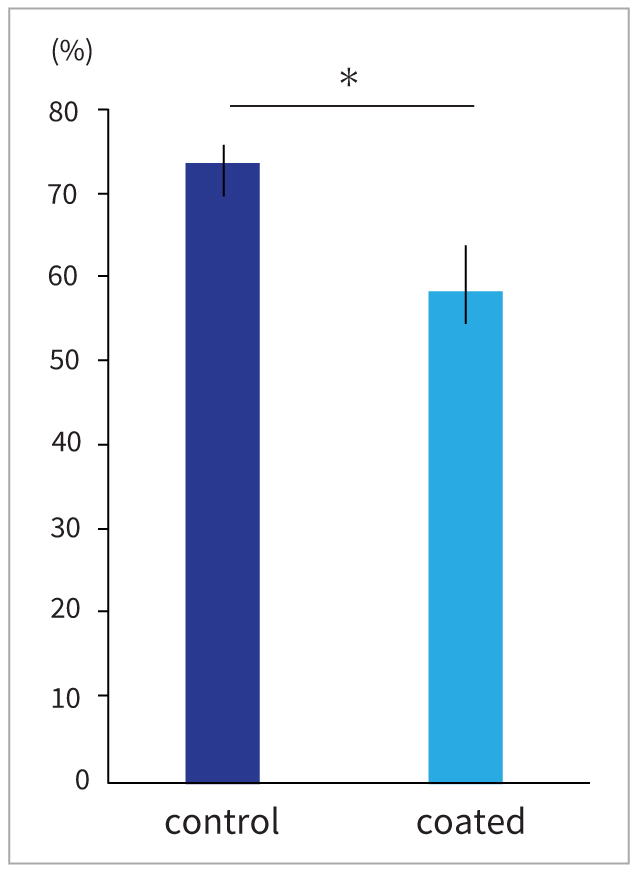
Adhesion rate on the test tube wall. Control: PRG Barrier Coat-uncoated group; coated: PRG Barrier Coat-coated group. Error bars indicate standard deviation. Asterisks indicate significant differences. Two-sample t-tests showed significant differences between the control and coated groups.

The application of PRG Barrier Coat resulted in a statistically significant decrease in the adhesion rate of the 12-h incubated biofilm to the inner surface of the glass tube (p < 0.05) (Fig 1). However, there was no statistically significant difference in the total amount of floating cells in the culture medium between the groups with and without PRG Barrier Coat (p = 0.377) (data not shown). Moreover, PRG Barrier Coat considerably reduced the amount of biofilm formed by *S. mutans* on the glass tube wall (p = 0.106; data not shown).

### Inhibitory Effects of PRG Barrier Coat on In-Vitro Biofilm Formation

**Fig 2:**
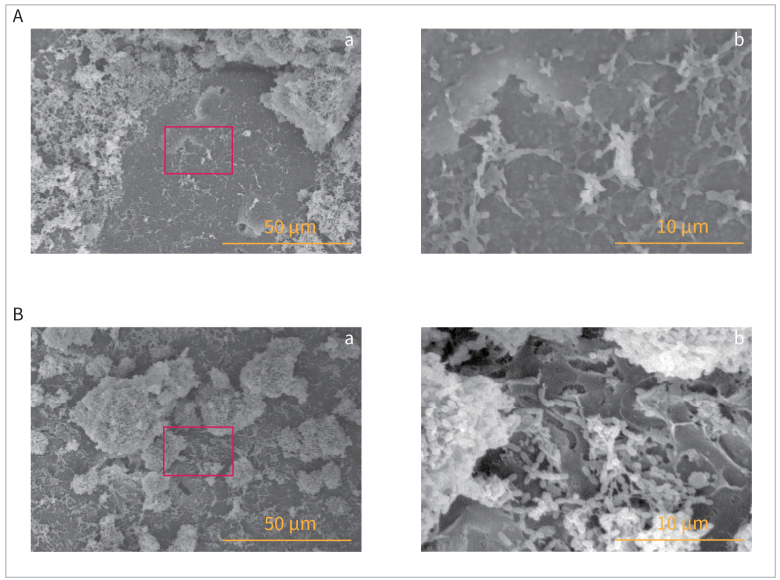
SEM images of in-vitro biofilm on HA disk. (a) and (b) show SEM images at 1000X and 5000X, respectively. (b) Magnified images of the area within the red rectangle (a). A: Biofilms incubated for 24 h on PRG Barrier Coat-uncoated HA disk (control). B: Biofilms incubated for 24 h on a PRG Barrier Coat-coated HA disk. HA, hydroxyapatite; SEM, scanning electron microscopy.

**Fig 3:**
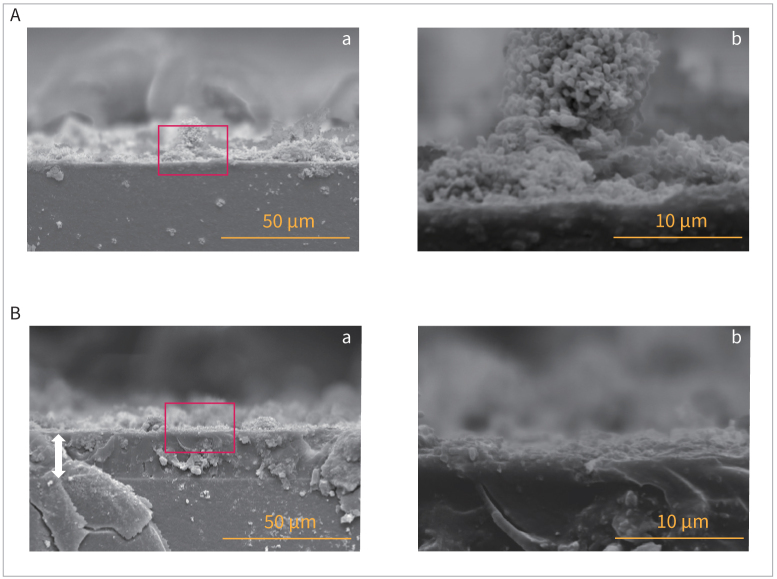
SEM images of in-vitro biofilm on HA disk cross-section. (a) and (b) show SEM images at 1000X and 5000X, respectively. The red rectangles in (a) indicate the areas enlarged in (b). A: Biofilms incubated for 24 h on PRG Barrier Coat-uncoated HA disk (control). B: Biofilms incubated for 24 h on a PRG Barrier Coat-coated HA disk. (a) The bidirectional arrows indicate the thickness of the PRG Barrier Coat applied to the HA disk. HA, hydroxyapatite; SEM, scanning electron microscopy.

In both the 12- and 24-h incubation samples, SEM images of the control group confirmed the formation of densely layered structures in *S. mutans* cells and the construction of layered filamentous networks that caused cells to adhere to each other, such that the external shape could not be fully recognised. SEM images of the smooth surface of a an HA disk incubated for 24 h are shown in Fig 2A. SEM images of the cross-section of the HA disk incubated for 24 h confirmed the presence of deposits on the disk’s surface, and clear biofilm formation was observed in the enlarged image (Fig 3A).

In contrast, in the PRG Barrier Coat-coated group, although clumps of *S. mutans* were observed, the bacterial layer was so thin that the surface to which the PRG Barrier Coat had been applied could be observed. The control group did not show any structures associated with adhesion (Fig 2B). In the SEM images of the cross section of the HA disk cultured for 24 h, a drug coating was confirmed on the disk surface, and there was no direct contact between the disk and the surface upon which bacteria adhered. In the enlarged image (b), almost no bacterial adhesion was observed on the coated surface (Fig 3B).

### Inhibitory Effects of S-PRG Barrier Coat on Expression Genes of *S. mutans*

**Fig 4:**
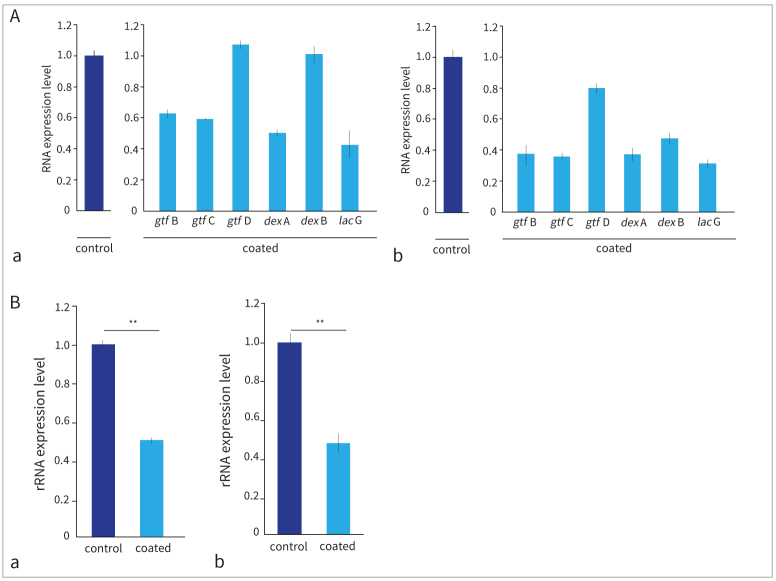
The gene expression level of biofilm. Control: PRG Barrier Coat-uncoated group; coated: PRG Barrier Coat-coated group. Error bars indicate standard deviation. Asterisks indicate statistically significant differences. A: Messenger RNA expression levels of *gtf*, *dex*, and *lac*G genes in the coated group, where the expression level of each mRNA gene in the control group is set to 1. (a) 12-h samples; biofilm prepared from incubation for 12 h. (b) 24-h samples. B: Ribosomal RNA expression levels of biofilm. (a) 12-h samples; the results of the analysis of biofilms prepared from incubation for 12 h, **p < 0.01; (b) 24-h samples, **p < 0.01.

Real-time RT-PCR was used to determine the mRNA expression levels of caries-related genes, including *gtf*s. Figure 4A shows the expression levels of each mRNA gene in the coated group (with the expression level of each mRNA gene in the control group set to 1). The expression of *gtf*B, *gtf*C, *dexA*, and *lac*G was suppressed in the biofilm obtained from the surface of the disk coated with PRG Barrier Coat. For the samples with 12-h biofilm formation, the expression levels of *gtf*D and *dex*B were approximately the same, regardless of the presence or absence of PRG Barrier Coat. However, when the biofilm matured after 24 h of incubation, the expression levels of *gtf*D and *dex*B decreased (Fig 4A). In both the 12- and 24-h samples, ribosomal RNA expression levels were suppressed by approximately half compared to those in the control samples (Fig 4B).

## Discussion

This study demonstrated that PRG Barrier Coat statistically significantly inhibited the adhesion of *S. mutans* and biofilm formation and suppressed the expression of caries-related genes. Real-time RT-PCR was used to compare the expression levels of caries-related genes. The expression levels, including those of *gtf*B, *gtf*C, and *gtf*D, decreased in the PRG Barrier Coat application group, suggesting that biofilm formation was decreased. Yamashita et al^22^ reported that *gtf*B, *gtf*C, and *gtf*D play important roles in smooth-surface caries formation using strains of *S. mutans* deficient in *gtf*B, *gtf*C, and *gtf*D. Therefore, suppression of the expression of these genes by PRG Barrier Coat was considered to contribute to the suppression of smooth-surface caries. The expression of *gtf*B and *gtf*C is controlled by *orf*1 and *orf*2, respectively,^18^ and further research is required to clarify the control mechanism in this material. Bowen et al^1^ stated that *gtf*B and *gtf*C gene expression increased in response to environmental acidification. The reduced expression of these genes can be explained by the effect of PRG Barrier Coat in regulating the pH of the local environment to weakly alkaline.

In addition, dextranase is produced extracellularly by *S. mutans*,^19^ and its optimum pH is 5.5, with activity reduced in a weakly alkaline environment.^8^ This enzyme works to transform glucan into modified glucan,^15^ and suppressing *dex*A expression reduces the amount of glucan biofilm. The expression levels of *gtf*D and *dex*B were approximately the same in samples with 12-h biofilm formation, regardless of PRG Barrier Coat. In contrast, when comparing the expression levels of *gtf*D and *dex*B in matured biofilms after 24 h of incubation, they decreased, along with those of other genes. The reason for this reactivity difference among the three *gft*Ds remains unclear. Therefore, further studies are warranted. The difference in reactivity between *dex*A and *dex*B may be attributed to their involvement in intracellular and extracellular metabolism, respectively.^16,19^ Based on the results of in-vitro adhesion inhibitory effects and real-time RT-PCR, it is suggested that PRG Barrier Coat does not inhibit *S. mutans* growth but inhibits adhesion factors. The microbe was present in a planktonic state in the test tube; however, in the actual oral cavity, *S. mutans* that do not adhere to the tooth surface are washed away by the self-cleaning action of saliva. Currently, there is no mouthwash containing S-PRG filler on the market, but it could be developed as a mouthwash or as an auxiliary product for coating materials and sealants.

The SEM images depicted a discernible shift in the attachment morphology of *S. mutans* in the biofilm between the control and PRG Barrier Coat application groups. Without PRG Barrier Coat, a dense bacterial layer with an adhesive structure was observed. However, in the presence of PRG Barrier Coat, the bacterial layer was thin and the adhesive structure was absent. The observed structural difference is believed to contribute to the reduction in the amount of *S. mutans* adhered to the surface. However, further investigation is needed to confirm whether this is directly attributed to the glucan produced. The SEM images further confirmed the presence of many voids between the bacterial layer on the PRG Barrier-Coat–coated disk. Within these voids, it was observed that no *S. mutans* adhered to the coated disk surface. This observation was not found in the uncoated group. Yamamoto et al^21^ also reported a reduction in biofilm formation in dentin samples coated with PRG Barrier Coat compared to uncoated samples. Our study demonstrated that this effect persisted for at least 24 h. Nomura et al^12^ reported that the presence of the S-PRG filler suppressed biofilm formation, as shown in confocal scanning laser microscopy, which is consistent with our findings. Additionally, real-time RT-PCR revealed a statistically significant reduction in the amount of 16S rRNA in the coated group, indicating similar results. Nomura et al^12^ also reported that the S-PRG eluate downregulates the operons involved in sugar metabolism and suppresses the proliferation of *S. mutans*, consistent with our observations that the expression of 16S rRNA was statistically significantly reduced by the application of this material containing the S-PRG filler.

Our absorption spectrophotometry experiments were conducted to investigate the effect of PRG Barrier Coat on bacterial adhesion, with The results showing a statistically significant reduction in adhesion rates with the application of this material. Clinical studies by Örtengren et al^14^ also reported a decrease in plaque accumulation and a tendency toward a reduction in *S. mutans* levels in subjects who used PRG Barrier Coat. This anti-adhesion effect was also observed in an experiment using S-PRG filler eluate.^24^ It is suggested that the S-PRG filler is responsible for this effect. In addition, Örtengren et al^10^ reported a decrease in the plaque index even on tooth surfaces to which PRB Barrier Coat were not applied, possibly due to the ion release of this material, including strontium, boron, and fluoride ions. However, the extent of this effect on the local oral environment is unknown, and further investigation is necessary.

This study has limitations that should be acknowledged. Firstly, due to its in-vitro nature, the study does not fully replicate the complex environment of the oral cavity in vivo. Therefore, the generalisability of the obtained results to clinical settings is uncertain. Additionally, while the study successfully demonstrated that PRG Barrier Coat suppressed biofilm formation by *S. mutans* through the inhibition of insoluble glucan synthase expression, it did not explore the duration of these effects on *S. mutans* adhesion, biofilm formation, or the expression of caries-related genes. Moreover, in clinical conditions, *S. mutans* typically forms biofilms in the presence of other pathogens or during mixed infections with *Streptococcus sobrinus* and other bacteria. These factors were not considered in the present study. Future research should address these limitations to provide a more comprehensive understanding of the effects of PRG Barrier Coat in clinical settings.

## Conclusion

This study suggests that tooth-surface coating materials, such as PRG Barrier Coat, have the potential to enhance caries prevention in dental practice. In this study, PRG Barrier Coat suppressed biofilm formation by *S. mutans* by inhibiting the expression of insoluble glucan synthase, which is involved in primary biofilm formation. This material also affected gene expression and altered the biofilm structure. To compensate for the limitations due to the in-vitro nature of this study, further studies under conditions more similar to the oral environment are encouraged.
